# Genome-Wide Analysis of the Aspartate Aminotransferase Family in *Brassica rapa* and the Role of *BraASP1* in Response to Nitrogen Starvation

**DOI:** 10.3390/ijms26041586

**Published:** 2025-02-13

**Authors:** Yan Liu, Zihan Gao, Chuang Liang, Yuting Wei, Yuge Li, Yan Zhang, Yaowei Zhang

**Affiliations:** 1 College of Horticulture and Landscape Architecture, Northeast Agricultural University, Harbin 150030, China; liuyanworld@126.com (Y.L.); bgxs9@163.com (Z.G.); lcyjq7@163.com (C.L.); wyt1236662023@163.com (Y.W.); 18236346679@163.com (Y.L.); 2 Key Laboratory of Biology and Genetic Improvement of Horticulture Crops (Northeast Region), Ministry of Agriculture and Rural Affairs, Northeast Agricultural University, Harbin 150030, China

**Keywords:** Chinese cabbage, synteny, phylogenetic analysis, gene expression

## Abstract

Nitrogen (N) is the most important fertilizer for increasing crop production, as it is absorbed by various N transporters and metabolized by a series of enzymes. Aspartate aminotransferase (ASP) facilitates the conversion of Glu to Asp for N storage. Chinese cabbage is a typical leafy vegetable that requires a large amount of N for growth. To investigate the functions of *BraASPs*, 10 members of the ASP gene family in *Brassica rapa* (*B. rapa*) were identified. Phylogenetic analysis and collinearity comparisons of ASP members among *B. rapa*, *Arabidopsis thaliana* (*A. thaliana*), *Oryza sativa* (*O. sativa*), *Brassica napus* (*B. napus*), and *Brassica oleracea* (*B. oleracea*) were conducted to examine evolutionary associations and genome duplication events across species. Multiple cis-acting elements associated with stress responses were identified in the promoters of *BraASPs*, suggesting their diverse roles. Members of *BraASPs* were expressed in roots, stems, flowers, siliques, and leaves, with the highest expression in leaves. Their expression levels increased rapidly at 3 h under low N conditions, peaked at 6 h, and returned to low levels at 24 h. Based on transcriptomic data, *BraASP1b* was identified as a candidate gene in *B. rapa* under low N stress, localized in the nucleus and cytoplasm. Overexpression of *BraASP1b* in *A. thaliana* resulted in a higher biomass than Col-0 under low N conditions.

## 1. Introduction

Nitrogen (N) is the most demanded essential nutrient for crops and serves as a critical fertilizer for achieving high production levels. Therefore, the substantial input of N fertilizers in agricultural production has reached 115–120 million tons annually, resulting in significant financial expenditure. However, the nitrogen use efficiency (NUE) in major crops worldwide ranges from only 30% to 50%, leading to higher costs, energy waste, environmental issues, and food safety concerns [1,2]. The NUEs of crops are influenced by a complex interplay of different crop species and environmental factors. Therefore, understanding the complex regulatory networks of N uptake and metabolism is essential for improving crop NUE and balancing N inputs with utilization [3,4].

Multiple forms of N could be absorbed by plants, including inorganic nitrates (NO_3_^−^) in high quantities and smaller amounts of ammonium (NH_4_^+^), as well as some organic nitrates such as amino acids, peptides, and proteins [5]. Ammonium (NH^4+^) is preferentially absorbed by plants through ammonium transporters (AMTs) and is directly assimilated for use. Studies have identified six *AtAMTs* in *A. thaliana* and 10 Os*AMTs* in rice, which belong to two gene families [3]. In contrast, four families of nitrate transporters are involved in the absorption of NO_3_^−^ by plants, including the nitrate transporter 1 (NRT1)/peptide transporter family (NPF), NRT2 family, chloride channel (CLC) family, and slowly activating anion channels [6]. Research has reported 53 NPF genes in *A. thaliana* and 93 in rice, which play significant roles in nitrate uptake from the soil, root-to-shoot transport, nitrate allocation among leaves, and seed developments [4]. The NRT2 members are primarily associated with a high-affinity transport system, comprising seven *AtNRT2* genes and four *OsNRT2* transporters, which are induced by nitrate deficiency and repressed under high nitrate levels [4,7]. Following the assimilation of N through various transporters, a range of enzymes are involved in N metabolism across multiple steps. Ammonium is directly utilized and reduced into glutamine by glutamine synthetase (GS), a key enzyme in N metabolism, existing in two forms: cytosolic GS1 and plastidial GS2 [8]. Subsequently, glutamate 2-oxoglutarate aminotransferase (GOGAT) participates in the conversion of glutamine to glutamate in the GS/GOGAT cycle. Additionally, pyruvate and glutamate are produced by aspartate aminotransferase (ASP) and alanine aminotransferase (AlaAT) in the cytosol for amino acid biosynthesis pathways [8,9]. Furthermore, there are two primary steps involved in the utilization of NO_3_^−^ in plants: it is first reduced to nitrite by nitrate reductase (NR) and then converted to ammonium by nitrite reductase within ammonium metabolic pathways [9,10].

ASP, also referred to as AAT, is recognized as a key enzyme in C and N metabolism, playing a vital role in the conversion of glutamate (Glu) to Asp during N assimilation in both animals and plants [11]. Various types of ASP members exist in *A. thaliana*, including cytosolic isozymes, plastidic forms, and prokaryotic types. Overexpression lines of *ASP2* in *Arabidopsis* exhibited no noticeable morphological changes but demonstrated increased sensitivity to *Botrytis cinerea* [12]. Additionally, overexpression of three *AAT* genes (*OsAAT1*, *OsAAT2*, and *OsAAT3*) in rice resulted in higher AAT activities in leaves, along with increased amino acid and protein contents in seeds, without any evident phenotypic alterations [13]. However, the AAT of *Medicago sativa* did not exhibit high NUE under either high or low N conditions in overexpression transgenic *Brassica napus* (*B. napus*) lines [14]. As previously noted, the functions of the AAT family members in N metabolism warrant further investigation in plants [10].

Chinese cabbage, a globally popular leafy vegetable, is particularly favored in Asian countries such as China, South Korea, and Japan. A significant amount of N is required throughout its growth stages, especially for the formation of the primary commercial organ, the leaf head [15]. However, the total NUE of vegetables is typically less than 20% [2,16], highlighting the need to explore the regulatory networks of N metabolism in these crops to enhance their NUEs. In our prior study, focusing on the transcriptomic analysis of sensitive and insensitive Chinese cabbage inbred lines, a candidate gene, *BraASP1*, was identified as responsive to low N stresses [17]. Therefore, the ASP family of *Brassica rapa* (*B. rapa*) was analyzed, including the characteristics of each ASP, as well as the evolutionary associations and collinearity of ASP members among *B. rapa*, *A. thaliana*, *Oryza sativa* (*O. sativa*), *B. napus*, and *B. oleracea.* Furthermore, the expression patterns of *BrASPs* were examined across different tissues and in response to low N stresses. Ultimately, the biomass and phenotypes of *BraASP1b* transgenic *A. thaliana* were investigated under low N treatments, indicating that *BraASP1b* plays a positive role in responding to low N conditions in *B. rapa*.

## 2. Results

### 2.1. The Phylogenetic Associations and Collinearity Analysis of ASP Members Among B. rapa, A. thaliana, O. sativa, B. napus, and B. oleracea

All 13 predicted members of the *ASP* gene family in *B. rapa* were sourced from the BRAD database and subsequently validated for the presence of ASP domains using PfamScan and InterPro. A total of 10 members of the ASP family were isolated from the *B. rapa* genome and renaming was conducted from Bra1a to Bra5b based on their homology with *A. thaliana* (Table S1). Additionally, detailed information regarding the *BraASP* members was compiled and presented in Table S2, which includes genome positions, lengths of coding sequences (CDS) and proteins, exon counts, molecular weights, and isoelectric points. The lengths of the CDS and protein sequences exhibited similarities, ranging from 1218 to 2625 bp, encoding proteins of 405 to 874 amino acids. Notably, *BraASP5a* emerged as the longest member of the *BraASP* family, extending up to 2625 bp with 20 exons, while the remaining genes demonstrated a relatively uniform length of approximately 1200 to 1400 bp.

**Figure 1:**
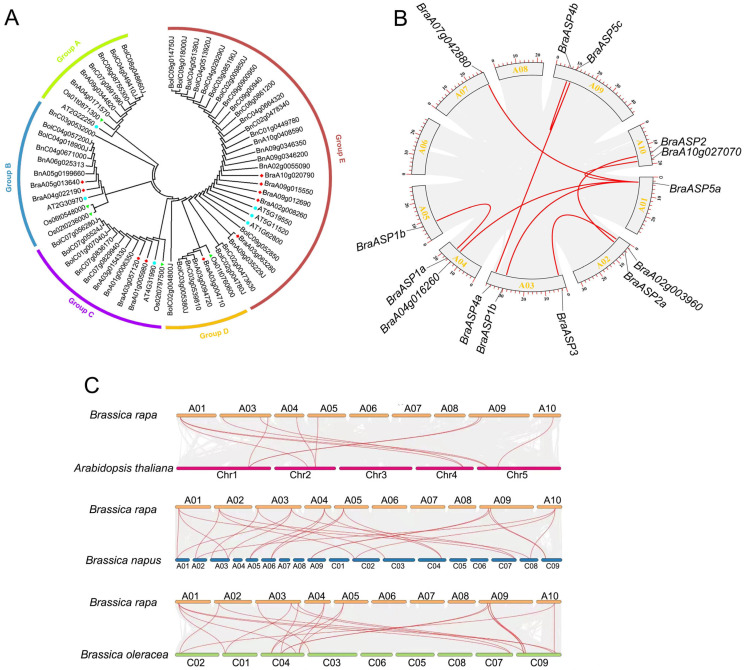
Whole phylogenetic tree and collinearity analysis of ASP members among *A. thaliana*, *O. sativa*, *B. rapa*, *B. oleracea,* and *B. nap*. (**A**) Whole phylogenetic tree of ASP protein among *A. thaliana*, *O. sativa*, *B. rapa*, *B. oleracea*, and *B. nap*; (**B**) The collinearity analysis of ASP members in *B. rapa*; (**C**) The collinearity analysis of ASP members among *A. thaliana*, *O. sativa*, *B. rapa*, *B. oleracea,* and *B. nap*.

The phylogenetic associations of the ASP proteins among *A. thaliana*, *B. rapa*, *B. napus*, *B. oleracea*, and *O. sativa* were analyzed and constructed utilizing the maximum likelihood method. This analysis included six ATASPs, 5 OsASPs, 10 BraASPs, 17 BolASPs, and 27 BnASPs, which were categorized into five subgroups labeled Group A through Group E (Figure 1A), with Group E being the largest, including 29 members. Most ASPs clustered primarily according to species rather than homologies. However, two copies of BraASP1 (BraA04g022190.3C and BraA05g013640.3C) were grouped together in Group B (Figure 1A).

Synteny analysis of the *BraASP* family genes was conducted to investigate repetitive events. A total of nine collinear pairs of *BraASPs* were identified in *B. rapa*, including pairs such as *BraASP1a* and *BraASP1b*; *BraASP2a* and *BraASP2b*; and *BraASP4a* and *BraASP4c*, along with *BraASP3b* and *BraA02g003960*; and four collinear pairs involving *BraASP5a*: *BraASP5b*, *BraA04g018650*, *BraA07g042860*, and *BraA10g027070* (Figure 1B). The majority of the collinear pairs corresponded to homologous genes. However, *BraA04g018650.3C*, *BraA07g042860.3C*, and *BraA10g027070.3C* encoded proteins belonging to the protein kinase superfamily. These findings indicate a higher frequency of repetitive events among ASPs in *B. rapa.*

Furthermore, collinearity analysis of ASP members between *B. rapa* and *A. thaliana*, *O. sativa*, *B. oleracea*, and *B. napus* was computed and displayed in Figure 1C. A significant number of collinear pairs were identified between *B. rapa* and other cruciferous plants, specifically 13 pairs with *A. thaliana*, 23 pairs with *B. oleracea*, and 24 pairs with *B. napus* (Figure 1C and Table S3).

### 2.2. The Cis-Acting Regulatory Elements of Promoters in BraASPs

The upstream 1500 bp sequence of each *BraASP* initiation codon was downloaded for the analysis of cis-acting regulatory elements, which were analyzed using the online tool PlantCARE. A total of 40 types of cis-acting elements were identified in the promoters of *BraASP* members, as depicted in Figure S1. The maximum number of cis-acting elements detected was 104 for the CAAT box, distributed among five *BraASP* members. Additionally, numerous stress-related cis-acting elements were found in the promoters of *BraASPs*, including ABRE for abscisic acid responsiveness, ARE for anaerobic induction, CGTCA-motif for MeJA responsiveness, MYB for abiotic stresses, MBS for drought-inducibility, and DRE for drought, salt, and low-temperature stresses, as well as LTR for low-temperature stresses, TC-rich repeats for defense and stress responsiveness, and TCA-element for salicylic acid responsiveness. These findings suggest that *BraASPs* may play significant roles in the plant’s response to stress.

### 2.3. The Structures and Expression Levels of BraASP Members

**Figure 2:**
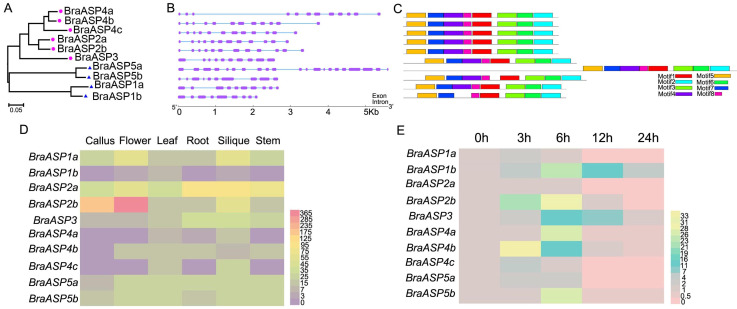
The structures and expression levels of *BraASP* members. (**A**) The phylogenetic tree of BraASP proteins; (**B**) The gene structures of *BraASP* members; (**C**) The protein motifs of BraASPs; (**D**) The expression levels of *BraASPs* in callus, flower, leaf, root, silique, stem; (**E**) The expression patterns of *BraASPs* under low N treatment for 0, 3, 6, 12, and 24 h.

The protein sequences of BraASPs were obtained from the BRAD database, and their phylogenetic relationships were analyzed using MEGA11 with the Neighbor-Joining method, clustering BraASPs into five subgroups based on homology (Figure 2A). Based on their CDS and genome sequences, the gene structures of *BraASP* members were analyzed using the Gene Structure Display Server, revealing that *BraASP* genes contained between 9 and 20 exons, with half of them having more than 12 exons (Figure 2B). Protein motifs of BraASPs were analyzed using MEME (Figure 2C and Figure S2), identifying a total of eight motifs (Table S4). Among these, seven motifs (motifs 1–3 and 5–7) were highly conserved, whereas motif 4 was absent in BraASP1b. These findings indicate that the gene structures and protein motifs of BraASP family members are highly conserved.

To investigate the potential molecular functions of *BraASPs*, expression levels were examined across different tissues and under low N stress (Figure 2D,E). *BraASP* gene expression was first assessed in roots, stems, leaves, flowers, siliques, and callus (Figure 2D), showing that most *BraASPs* were highly expressed in leaves, with the lowest expression observed in callus. *BraASP1a*, *BraASP2a*, *BraASP2b*, *BraASP5a*, and *BraASP5b* exhibited high expression across all detected tissues, whereas *BraASP1b* had low expression in all tissues except leaves. *BraASP4a*, *BraASP4b*, and *BraASP4c* were induced in leaves and siliques. Additionally, *BraASP* expression under low N stress was analyzed by qPCR (Figure 2E). Most *BraASP* genes were markedly induced from 3 h after low N treatment, particularly *BraASP2b* and *BraASP4b*, which showed 21-fold and 33-fold increases, respectively, peaking at 6 h when *BraASP2b* reached a 30.8-fold increase. *BraASP1b* maintained high expression levels under low N conditions from 3 h to 24 h.

### 2.4. The Expressions of BraASPs in Sensitive and Insensitive Chinese Cabbage Inbred Lines Under Low N Stresses

**Figure 3:**
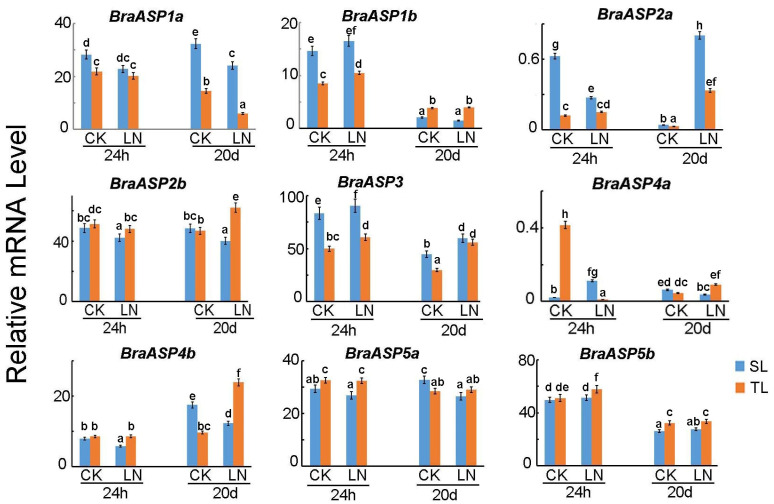
The expression levels of *BraASP* members in sensitive (SL) and tolerant inbred lines (TL) of Chinese cabbage in response to low N under short (24 h) and long (20d) treatments by transcriptome analysis. Data are means ± SD with three biological repetitions; different letters represent significant differences (*p* < 0.05) between all values.

A previous study identified sensitive (SL) and tolerant (TL) inbred lines of Chinese cabbage based on their responses to low N stress. Differentially expressed genes (DEGs) were analyzed through transcriptomic analysis over short (24 h) and long (20-day) treatment durations [17]. Most *BraASP* (9 of 10) members, except for *BraASP4c*, were identified from the RNA-Seq data, as shown in Figure 3. During the short-term low N stress, expression levels of *BraASP1a*, *BraASP1b*, *BraASP2a*, and *BraASP3* were higher in SL compared to TL, while *BraASP2b*, *BraASP4b*, *BraASP5a*, and *BraASP5b* displayed lower expression in SL than in TL. For the long-term stress analysis, the expression patterns of most *BraASP* genes were consistent with those observed in the short-term analysis, with the exception of *BraASP1b*, which was significantly inhibited by low N and exhibited higher expression in TL compared to SL. In comparing the long-term and short-term conditions, four *BraASP* genes—*BraASP1b*, *BraASP2a*, *BraASP4a*, and *BraASP5b*—showed decreased expression, whereas three members—*BraASP2b*, *BraASP3*, and *BraASP4*—were induced.

### 2.5. The Overexpression of BraASP1b in A. thaliana Under Low N Treatments

**Figure 4:**
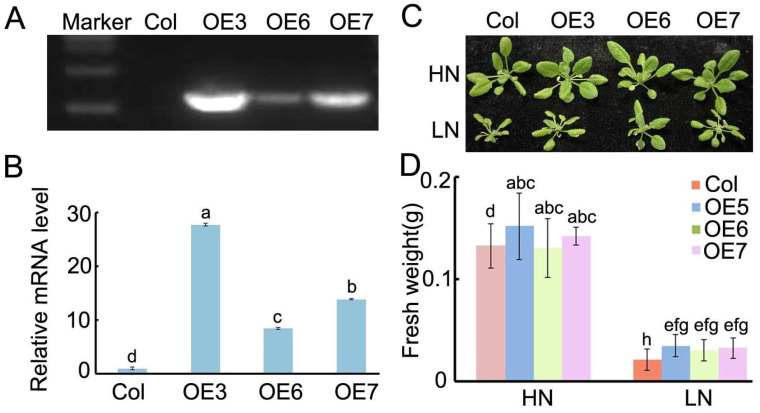
The identification and phenotypes of overexpressed *BraASP1b* transgenic *A. thaliana* lines under high and low N conditions. (**A**) The identification of overexpressed *BraASP1b* transgenic *A. thaliana* lines by PCR; (**B**) The expression levels of *BraASP1b* in overexpressed transgenic *A. thaliana* lines; (**C**) The phenotypes of overexpressed *BraASP1b* transgenic *A. thaliana* lines under high and low N conditions; (**D**) The fresh weights of *BraASP1b* transgenic *A. thaliana* lines under high and low N conditions. Data are means ± SD with three biological repetitions; different letters represent significant differences (*p* < 0.05) between all values.

A previous study identified *BraASP1b* as a candidate gene in Chinese cabbage in response to low N conditions [17]. To further investigate its expression characteristics, *BraASP1b* was cloned and transfected into *A. thaliana*. Three positive transgenic lines (T3 progenies) were obtained following hygromycin screening and designated as OE5, OE6, and OE7. Specific primers for *BraASP1b* were designed to identify the transgenic lines using PCR, with Col-0 serving as a negative control, as depicted in Figure 4A. Subsequently, the expression levels of *BraASP1b* in the overexpressing lines were quantified using qPCR, revealing that, compared to Col-0, OE5, OE6, and OE7 exhibited upregulation by factors of 27.8, 8.5, and 13.8, respectively (Figure 4B).

The *BraASP1b* transgenic lines OE5, OE6, and OE7 were subjected to low N conditions for a duration of 20 days, with high N serving as a control for phenotypic investigations (Figure 4C). Under high N conditions, OE5, OE6, and OE7 were found to be larger than Col-0. Although all plants exhibited a reduced size under low N, these three overexpressing transgenic lines remained larger than Col-0 (Figure 4C). Further measurements of the fresh weights of each plant were taken, aligning with the observed phenotypes of the *BraASP1b* transgenic lines (Figure 4D). These results indicate that the overexpression of *BraASP1b* in *A. thaliana* enhances biomass under both high and low N treatments.

### 2.6. The Subcellular Localization of BraASP1b-GFP in Nicotiana benthamiana (N. benthamiana)

**Figure 5:**
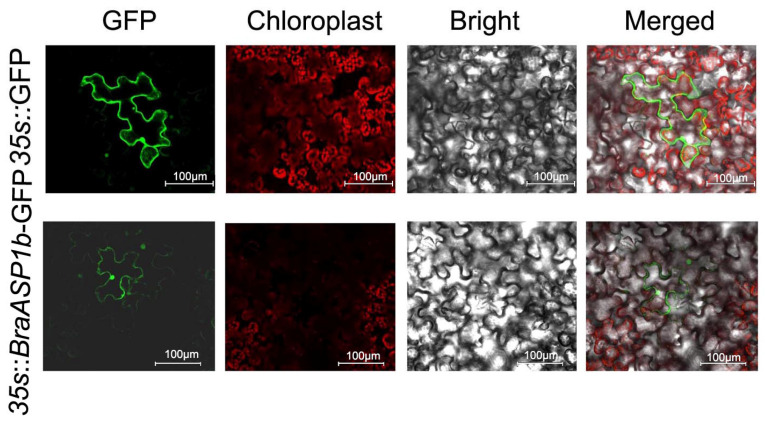
The subcellular localization of *35S*::*BraASP1b*-GFP in *N. benthamiana*.

Additionally, the subcellular localization of *35S*::*BraASP1b*-GFP was examined in *N. benthamiana*, as illustrated in Figure 5. The results indicated that BraASP1b-GFP is localized in both the cytoplasm and the nucleus, while *35S*::GFP served as a control, demonstrating localization in both compartments.

### 2.7. The Physiological Characteristics of BraASP1b Overexpressed Transgenic A. thaliana Lines Under Low N Stresses

**Figure 6:**
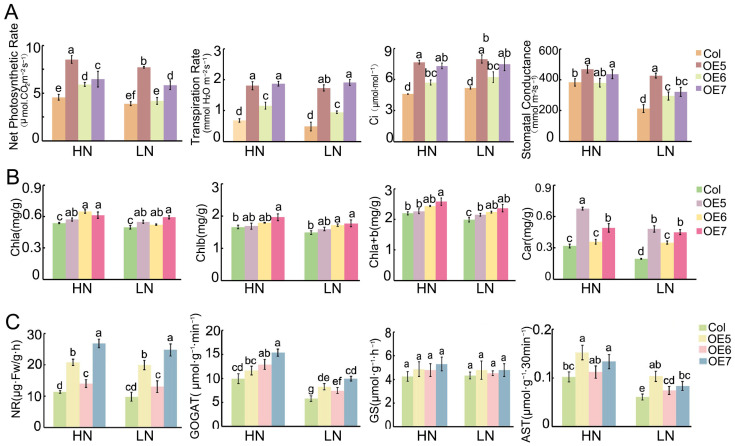
The physiological characteristics of *BraASP1b* overexpressed transgenic *A. thaliana* lines under high and low N conditions. (**A**) The photosynthetic characteristics of net photosynthetic rate, respiration rate, intercellular carbon dioxide concentration (Ci) and stomatal conductance in Col-0 and *BraASP1b* overexpressed transgenic *A. thaliana* under high and low N; (**B**) The contents of chlorophylls and carotenoids in Col-0 and *BraASP1b* overexpressed transgenic *A. thaliana* under high and low N; (**C**) The activities of N-metabolism-related enzymes in Col-0 and overexpressed *BraASP1b* transgenic *A. thaliana* lines under high and low N conditions. Data are means ± SD with three biological repetitions; different letters represent significant differences (*p* < 0.05) between all values.

Furthermore, the physiological characteristics of *BraASP1b* overexpressed transgenic *A. thaliana* and Col-0 were examined under both high and low N treatments, including photosynthetic characteristics, chlorophyll and carotenoid contents, and enzyme activities involved in N metabolism. Initially, the net photosynthetic rate, transpiration rate, intercellular carbon dioxide concentration, and stomatal conductance were measured after 20 days of low N conditions (Figure 6). Under HN conditions, all these metrics for Col-0 were significantly lower than those for the *BraASP1b* overexpressed transgenic *A. thaliana* lines. When subjected to low N treatment, all the photosynthetic metrics decreased; however, the *BraASP1b* transgenic *A. thaliana* lines exhibited higher values than Col-0 (Figure 6A), with line OE7 showing the highest performance. Subsequently, the contents of chlorophyll a (Chla), chlorophyll b (Chlb), and carotenoids were measured. The results indicated that the Chla/b contents were slightly higher in the *BraASP1b* transgenic lines compared to Col-0 under both HN and LN conditions (Figure 6B). The carotenoid levels in lines OE5 and OE7 were significantly higher than in Col-0 under HN (Figure 6B), and although these levels decreased under LN, they remained higher in the *BraASP1b* overexpressed transgenic lines compared to Col-0.

The activities of the enzymes NR, GOGAT, GS, and ASP were also measured in both the *BraASP1b* overexpressed transgenic *A. thaliana* lines and Col-0, all of which play key roles in N metabolism. These enzyme activities were suppressed under low N treatments, with GOGAT showing the most substantial reduction. The activities of NR and GOGAT remained higher in the transgenic *A. thaliana* lines compared to Col-0 under both HN and LN treatments; however, there were no significant differences in GS activity between the two lines (Figure 6C). Due to the overexpression of *BraASP1b* in the transgenic *A. thaliana*, the enzyme activities of ASP were elevated compared to Col-0 under both HN and LN conditions.

### 2.8. The Expression Levels of Abiotic Stress and N-Metabolism-Related Genes in Transgenic A. thaliana Lines of Overexpressed BraASP1b Under Low N Stresses

**Figure 7:**
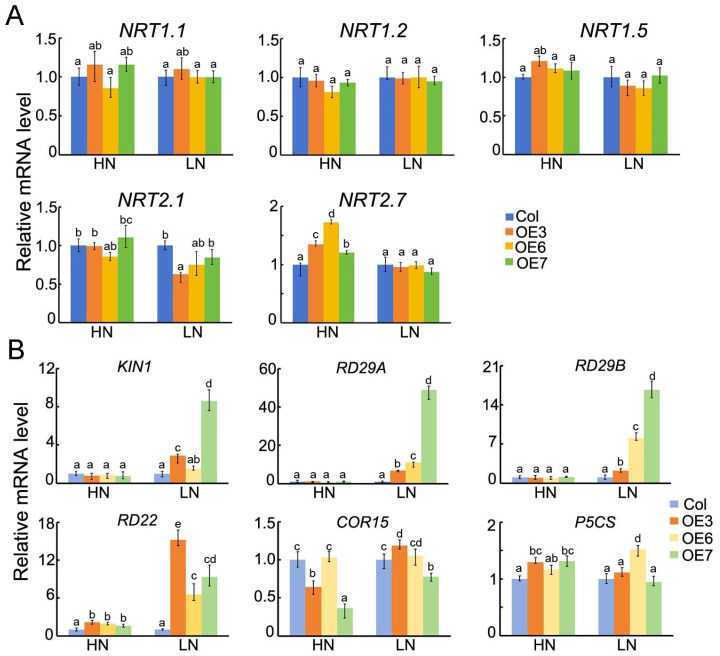
The expression levels of abiotic stress and N-metabolism-related genes in overexpressed *BraASP1b A. thaliana* lines under low N stresses. (**A**) The expression levels of N-transporter genes in overexpressed *BraASP1b* transgenic lines under low N stresses; (**B**) The expression levels of abiotic stress-related genes in overexpressed *BraASP1b A. thaliana* lines under low N stresses. Data are means ± SD with three biological repetitions; different letters represent significant differences (*p* < 0.05) between all values.

The results above indicate that *BraASP1b* overexpression enhances tolerance to low N conditions in transgenic *A. thaliana*. To investigate the involvement of *BraASP1b* in N-metabolism pathways, the transcription levels of *NRT1.1*, *NRT1.2*, *NRT1.5*, *NRT2.1*, and *NRT2.7* were analyzed under low N conditions. No significant differences were observed between transgenic lines and Col-0 under either high or low N conditions (Figure 7A).

Since low N stress is considered an abiotic stressor, several abiotic stress-related genes, including *KIN1*, *RD29A*, *RD29B*, *RD22*, *COR15*, and *P5CS*, were analyzed under low N conditions. The expression of *KIN1*, *RD29A*, and *RD29B* was significantly upregulated in *BraASP1b* transgenic lines under low N stress (Figure 7B). Among these, OE3 exhibited the highest expression levels, with fold increases of 8.6, 19, and 15.2, respectively, followed by OE7 and OE6. Additionally, *RD22* was upregulated in *BraASP1b* transgenic lines under both high and low N conditions, with even higher expression under low N. In contrast, no significant differences were observed in the expression of *COR15* and *P5CS*.

## 3. Discussion

ASP family members have been identified in various plant species, including *Panicum miliaceum* and *Neoporphyra* haitanensis, as well as the model dicot *A. thaliana* and monocot *O. sativa* [10]. In this study, the ASP gene family of *B. rapa* (*BraASP1* to *BraASP5*) was identified and characterized based on homology with *A. thaliana*, comprising 10 members. Structural analysis of their CDS, proteins, exons, molecular weights, and isoelectric points revealed high similarity among BraASP members, particularly in terms of sequence length, gene structure, and protein motifs. In *A. thaliana*, five classical ASP members belong to Class I [12]. Phylogenetic and collinearity analyses among *B. rapa*, *A. thaliana*, *O. sativa*, *B. napus*, and *B. oleracea* showed that ASP1 and ASP5 members in *B. rapa*, *A. thaliana*, *B. napus*, and *B. oleracea* clustered together. BraASP1a and BraASP1b, as well as BraASP5a and BraASP5b, were more closely related to AtASP1 and AtASP5 than to ASP members from *B. napus* and *B. oleracea*. BraASP3 exhibited greater similarity to Os01t0760600 rather than AtASP3, whereas AtASP3, AtASP2, and AtASP4 formed a separate cluster, grouping BraASP2a, BraASP2b, BraASP4a, and BraASP4b together. Collinearity analysis of *BraASPs* genes in the *B. rapa* genome identified nine collinear gene pairs. Additional collinearity associations were observed between *B. rapa* and *A. thaliana*, *B. napus*, and *B. oleracea*, with 13, 24, and 23 collinear gene pairs, respectively. These findings suggest frequent duplication events in *B. rapa* and other cruciferous species, but not in rice.

N is absorbed through various transporters and assimilated into Glu and Gln via the glutamine synthetase/glutamate synthase (GS/GOGAT) cycle. ASPs then convert these amino acids into Asp and asparagine (Asn), which serve as N transport and storage molecules [7,18]. To explore the molecular functions of *BraASP* genes, expression patterns were examined in different tissues and under low N stress in *B. rapa* seedlings. All *BraASPs* genes exhibited high expression levels in leaves. Notably, *BraASP1b* was highly expressed in all detected tissues. Most *BraASP* genes were significantly induced at 6 h after low N treatment, indicating early responsiveness to N deficiency. Furthermore, *BraASP1b* showed sustained induction under low N conditions, suggesting a crucial role in N metabolism.

Previous research identified *BraASP1b* as a candidate gene in a co-expression network analysis of different *B. rapa* seedlings responses to low N stress [17]. Overexpression of *ASP2* in *A. thaliana* did not alter plant morphology but increased sensitivity to *Botrytis cinerea* [12]. In this study, *BraASP1b* was cloned based on the *B. rapa* genome sequence and introduced into *A. thaliana* Col-0. Three positive transgenic lines (OE5, OE6, OE7) were generated and exposed to low N stress. Each transgenic line exhibited greater biomass accumulation than Col-0 under both high and low N conditions (Figure 4C,D). These results indicate that *BraASP1b* overexpression enhances biomass production under varying N conditions. ASP isoenzymes localize to different subcellular compartments, including mitochondria, chloroplasts/plastids, and the cytosol [19]. Subcellular localization analysis in *N. benthamiana* revealed that *BraASP1b* is primarily localized in the cytoplasm and nucleus (Figure 5).

Next, the physiological characteristics of *BraASP1b*-overexpressing transgenic *A. thaliana* and Col-0 were analyzed, including photosynthetic traits, chlorophyll and carotenoid content, and enzyme activities involved in N metabolism. N supply is essential for photosynthesis through photosynthesis, photorespiration, and respiration [20]. Photosynthetic characteristics of Chinese cabbage seedlings decreased under low N stress; however, they remained higher in low N-insensitive inbred lines than in sensitive lines [17]. In this study, each detected photosynthetic characteristic in *BraASP1b*-overexpressing transgenic *A. thaliana* was higher than in Col-0, suggesting enhanced photosynthetic efficiency in transgenic lines. Various enzymes participate in plant N metabolism. NR converts NO_3_^−^ to NO_2_^−^, which is subsequently reduced to NH_4_^−^. Ammonium is directly assimilated via the GS/GOGAT pathway and ultimately metabolized into aspartate by ASPs for storage [9]. Among these, GOGAT activity exhibited the most significant changes in *BraASP1b*-overexpressing transgenic *A. thaliana* under low N. Moreover, NR, GOGAT, and ASP activities were higher in transgenic lines than in Col-0 under both high N and low N conditions, contributing to enhanced N utilization efficiency in *BraASP1b* transgenic lines.

To investigate the regulatory pathways underlying biomass improvements in *BraASP1b* transgenic *A. thaliana*, the expression levels of N-transporter genes were analyzed under low N stress. Surprisingly, *NRT1.1*, *NRT1.2*, *NRT1.5*, *NRT2.1*, and *NRT2.7* showed no significant differences between transgenic *A. thaliana* lines and Col-0 under low N, indicating that *BraASP1b* had no major effect on transporter gene expression. This may be due to ASP’s primary role in N assimilation rather than absorption and transport. In contrast, multiple studies have shown that asparagine synthetase, particularly *Asn synthetase 1*, plays a key role in stress responses and N metabolism. Since asparagine biosynthesis is crucial for N metabolism [21], stress can impair plant nutrient metabolism, particularly N metabolism. The N utilization of the *rst1* loss-of-function mutant in *rice salt tolerant1* improved due to increased *Asn synthetase 1* expression, which prevented excessive NH_4_^+^ accumulation [22]. Similarly, *Asn synthetase 2* mutants exhibited reduced salt stress tolerance and impaired N assimilation, whereas low salt stress induced *Asn synthetase 1* expression [23]. Additionally, three *ZmAS* genes (*ZmAS1*, *ZmAS2*, and *ZmAS3*) were differentially regulated under post-silking drought stress in maize [24]. Drought stress also reduced total N levels in maize, but *ZmAS3* and *ZmAS4* were significantly induced [25]. Moreover, overexpression of pepper *Asn synthetase 1* in *A. thaliana* enhanced resistance to *Pst* DC3000 and *Hyaloperonospora arabidopsidis* [21]. Given the association between N metabolism and stress responses, we also examined the expression of abiotic stress-related genes in *BraASP1b* transgenic *A. thaliana*. *KIN1*, *RD29A*, *RD29B*, and *RD22* are involved in abiotic stress responses via the ABA-dependent pathway, particularly in cold, drought, and salt stress [26,27,28]. *COR15* functions in cold and freezing tolerance [29], while *P5CS* participates in proline biosynthesis and serves as a stress marker [30]. Notably, *RD29A*, *RD29B*, and *RD22* were significantly induced by low N in transgenic *A. thaliana* lines, whereas *COR15* and *P5CS* showed no differences. These findings suggest that *BraASP1b* transgenic *A. thaliana* enhances N utilization by increasing the activity of N assimilation-related enzymes.

## 4. Materials and Methods

### 4.1. Plant Materials

The inbred lines C14 and A213 of Chinese cabbage were cultivated in a greenhouse under 16 h of light (28 °C) and 8 h of darkness (18 °C) until the development of four leaves. The seedlings were subjected to high N (15 mmol·L^−1^ NO_3_^−^) and low N (5 mmol·L^−1^ NO_3_^−^) treatments, as described in our previous study [17], for durations of 0, 3, 6, 12, and 24 h. The remaining seedlings underwent vernalization under 16 h of light (8 °C) and 8 h of darkness (4 °C) for 21 days before being cultured under normal conditions until flowering. Tissues from the root, stem, leaf, flower, and siliques were harvested, with three biological replicates collected for each tissue type.

### 4.2. Identification of ASP in Chinese Cabbage

All the ASP members of Chinese cabbage were identified using the BRAD database (http://brassicadb.cn/#/, accessed on 1 July 2023) and isolated from version 3.0 of the *B. rapa* genome. Each member was subsequently searched in the NCBI database (https://blast.ncbi.nlm.nih.gov, accessed on 10 July 2023) to identify the accessions PLN02397 and pfam00155, associated with aminotransferase classes I and II. The proteins were further confirmed using online tools such as InterPro (http://www.ebi.ac.uk/interpro/, accessed on 12 July 2023) and PfamScan (https://www.ebi.ac.uk/Tools/pfa/pfamscan/, accessed on 12 July 2023).

### 4.3. The Cis-Acting Elements in Promoters, Gene Structures, and Collinearity Analysis

The 1500 bp upstream of each initiation codon was retrieved from the BRAD database, presumed to represent the promoters of the *BraASP* genes. All sequences were analyzed using the PlantCARE online tool (http://bioinformatics.psb.ugent.be/webtools/plantcare/html/, accessed on 15 July 2023).

The CDS and genomic sequences of *BraASP* members were downloaded from the BRAD database, with gene structures constructed using the online tool Gene Structure Display Server (http://gsds.cbi.pku.edu.cn, accessed on 18 July 2023).

The genomic and coding sequences of *A. thaliana*, rice, *B. napus*, and *B. oleracea* were acquired from Tair (https://www.arabidopsis.org/, accessed on 15 July 2023), Gramene databases (http://rice.uga.edu/, accessed on 15 July 2023), *Brassica napus* and *Brassica oleracea* (http://brassicadb.cn/#/, accessed on 18 July 2023). The synteny associations of *BraASP* among *A. thalian*, rice, *B. napus*, and *B. oleracea* were analyzed by MCScanX [31], associated with TBtools (version 2.080) [32].

### 4.4. Phylogenetic Tree and Motifs Analysis of ASP Protein

The protein sequences of ASPs from *A. thaliana*, rice, *B. napus*, *B. oleracea*, and Chinese cabbage were compiled. Phylogenetic trees were constructed using the MAGE11 software through maximum likelihood methods. The protein sequences of BraASPs were input into the MEME online tool (https://meme-suite.org/meme/, accessed on 30 July 2023) to identify eight conserved motifs within each BraASP.

### 4.5. Total RNA Extraction and Real-Time PCR Analysis

The plants of *A. thaliana* and Chinese cabbage were harvested, and the total RNA was extracted using the Plant RNA Extraction Kit (Omega, Ya Anda Biotechnology Co., Ltd., Beijing, China). The RNA was then reverse-transcribed to cDNA using the HiScript III 1st Strand cDNA Synthesis Kit (Vazyme Biotech Co., Ltd., Nanjing, China). A total volume of 20 microliters was used for real-time PCR, which included 1 μL of specific primers, 3 μL of diluted samples, and 7.5 μL of ChamQ SYBR qPCR Master Mix (Vazyme Biotech Co., Ltd., Nanjing, China) on a qTower3G (Analytik Jena, Jena, Germany) under 40 cycles, with *Tubulin* serving as the internal reference, as outlined in Table S5.

### 4.6. Plasmid Construction and Plant Transformation

The sequence of *BraASP1b* was successfully synthesized following codon optimization and the addition of nucleotides at the 3′ UTR, including TGAATT and GGTGACC (BstEII), as depicted in Figure S3 (Azentz, Suzhou, China). The fragments of *BraASP1b* were constructed into pCAMBIA-1302 via homologous recombination using the ClonExpress II One Step Cloning Kit (Vazyme Biotech Co., Ltd., China). Recombinant clones were screened using kanamycin and sequenced with specific primers (*BraASP1*-ID) listed as follows: forward primer: 5′-ATGGCTATGATGGCTAGAAC-3′; reverse primer: 5′-AGACTTAGTAACCTCATGGA-3′. The recombinant plasmids were subsequently transformed into Agrobacterium GV3101 strains using an Eppendorf Eporator (Eppendorf, Hamburg, Germany). The Agrobacterium was then transformed into *A. thaliana* Col-0 via the floral-dip method [33] and into *N*. *benthamiana* using a semi-injection method [34]. Transgenic *A. thaliana* were screened using hygromycin to obtain T3 progeny for phenotype observations, enzyme activity detections, chlorophyll pigment analysis, and gene expression levels under low N (5 mmol·L^−1^ NO_3_^−^) conditions, with high N (15 mmol·L^−1^ NO_3_^−^) serving as a control. The *BraASP1*-GFP fusion protein was transferred into *N*. *benthamiana* for observation using Nikon A1R/A1 confocal microscopy (Nikon Corporation, Tokyo, Japan).

### 4.7. Physiological Assays

Col-0 and *BraASP1b* overexpressing *A. thaliana* lines were treated and harvested as previously described. The net photosynthetic rate, transpiration rate, intercellular carbon dioxide concentration (Ci), and stomatal conductance of the leaves were measured using a LI-6400 (LI-COR, Lincoln, NE, USA) platform. The enzymatic activities of NR, GS, and ASP were measured as previously described [17] and GOGAT [35]. Each experiment was repeated with three biological replicates.

## 5. Conclusions

N is essential for the growth of Chinese cabbage, necessitating an investigation into the function of *ASPs* in *B. rapa*. In this study, 10 *BraASP* genes were identified across the *B. rapa* genome, exhibiting similarities in coding sequence length, gene structure, and protein motifs. Phylogenetic analysis of ASP proteins in *B. rapa*, *A. thaliana*, *O. sativa*, *B. napus*, and *B.oleracea* revealed that *BraASPs* genes clustered with their homologs in *A. thaliana*, with closer evolutionary associations to *B. napus* and *B. oleracea*. Collinearity analysis of *BraASP* genes in the *B. rapa* genome identified nine collinear gene pairs, whereas comparative analysis revealed 13, 24, and 23 collinearity pairs between *B. rapa* and *A. thaliana*, *B. napus*, and *B. oleracea*, respectively, indicating frequent gene duplication events among these species. To examine the molecular functions of *BraASP* genes, their expression patterns were analyzed across various tissues, including roots, stems, leaves, flowers, siliques, and callus. Most *BraASP* genes were induced under low N conditions, with *BraASP1b* exhibiting consistent induction across all time points. Transcriptomic analysis showed that *BraASP1b* expression was suppressed during short-term low N treatment in low N-insensitive *B. rapa* lines but was induced under long-term low N conditions compared to sensitive lines. To further investigate *BraASP1b* function, transgenic *A. thaliana* lines overexpressing *BraASP1b* were generated. These lines exhibited increased biomass compared to Col-0 under both high and low N conditions, with higher enzyme activities of NR and GOGAT. Additionally, *BraASP1b* overexpression resulted in an increased net photosynthetic rate, transpiration rate, intercellular carbon dioxide concentration, stomatal conductance, and the activities of NR, GOGAT, and GS compared to Col-0. However, no significant changes were observed in the expression of nitrate transporter genes (*NRT1.1*, *NRT1.2*, *NRT1.5*, *NRT2.1*, and *NRT2.7*) between Col-0 and *BraASP1b* transgenic *Arabidopsis* lines. In contrast, several abiotic stress-related genes (*RD29A*, *RD29B*, and *RD22*) were significantly upregulated under low N conditions in *BraASP1b* transgenic *A. thaliana*, suggesting a role for *BraASP1b* in improving N utilization and stress response mechanisms.

## Data Availability

The original contributions presented in this study are included in the article/Supplementary Material. Further inquiries can be directed to the corresponding author(s).

## Supplementary Materials

The following supporting information can be downloaded at: https://www.mdpi.com/article/10.3390/ijms26041586/s1.
