# Supplementary material for: Genome-Wide Analysis of the Aspartate Aminotransferase Family in Brassica rapa and the Role of BraASP1 in Response to Nitrogen Starvation
Source: Int J Mol Sci. 2025 Feb 13;26(4):1586. doi: 10.3390/ijms26041586 (PMC11855856; doi:10.3390/ijms26041586)
Supplement: Supplementary file 1 [file ijms-26-01586-s001.zip › ijms-3438334-supplementary.pdf]

Table S1 The homologies of *BraASP* in *A. thaliana*

Gene ID	*A. thaliana* homology gene	Rename
BraA04g022190.3C	AT2G30970	BraASP1a
BraA05g013640.3C	AT2G30970	BraASP1b
BraA10g020790.3C	AT5G19550	BraASP2
BraA02g003960.3C	AT5G11520	BraASP3a
BraA03g004710.3C	AT5G11520	BraASP3b
BraA03g063280.3C	AT1G62800	BraASP4a
BraA09g012690.3C	AT1G62800	BraASP4b
BraA09g015550.3C	AT1G62800	BraASP4c
BraA01g005980.3C	AT4G31990	BraASP5a
BraA03g057120.3C	AT4G31990	BraASP5b

Table S2 The messages of BraASP members

Gene name	Gene ID	Genome Position	CDS (bp)	Exon	Protein (aa)	MW (Da)	Isoelectric Point
BraASP1a	BraA04g022190.3C	A04:16481267..16484547	1281	10	426	47585.29	8.38
BraASP1b	BraA05g013640.3C	A05:7640149..7642870	1275	9	424	47291.16	8.57
BraASP2a	BraA02g008260.3C	A02:3937927..3941463	1281	12	405	44133.55	6.84
BraASP2b	BraA10g020790.3C	A10:14970462..14974409	1281	12	405	44217.58	6.6
BraASP3	BraA03g004710.3C	A03:2035149..2036868	1359	10	452	49288.66	9.34
BraASP4a	BraA03g063280.3C	A03:35819121..35825110	1218	12	405	44744.37	6.13
BraASP4b	BraA09g012690.3C	A09:7437311..7441683	1218	12	405	44574.32	6.07
BraASP4c	BraA09g015550.3C	A09:9265171..9268930	1218	12	405	44654.02	7.67
BraASP5a	BraA01g005980.3C	A01:2874843..2875702	2625	20	874	96404.64	8.78
BraASP5b	BraA03g057120.3C	A03:29989638..29992896	1437	10	478	52989	8.73

Table S3 The collinearity analysis of ASP members between *B. rapa* and other species.

B. rapa	*A. thaliana*	*B. rapa*	*B. oleracea*	*B. rapa*	*B. nap*
BraA01g005980	AT2G25220	BraA01g005980	BolC01g007040	BraA01g005980	BnA01g0006950
BraA01g005980	AT4G31990	BraA01g005980	BolC04g052880	BraA03g057120	BnA01g0006950
BraA01g005980	AT5G11020	BraA01g005980	BolC07g056280	BraA02g008260	BnA02g0055090
BraA02g008260	AT5G19550	BraA01g005980	BolC09g061680	BraA10g020790	BnA02g0055090
BraA03g063280	AT1G62800	BraA02g008260	BolC02g009850	BraA01g005980	BnA03g0154330
BraA03g057120	AT4G31990	BraA02g008260	BolC09g052850	BraA03g004710	BnA03g0094720
BraA03g004710	AT5G11520	BraA03g057120	BolC01g007040	BraA03g057120	BnA03g0154330
BraA04g016260	AT2G22250	BraA03g004710	BolC03g005380	BraA04g022190	BnA05g0199660
BraA04g022190	AT2G30970	BraA03g063280	BolC04g029290	BraA05g013640	BnA05g0199660
BraA05g013640	AT2G30970	BraA03g057120	BolC07g056280	BraA04g022190	BnA06g0253130
BraA09g012690	AT1G62800	BraA03g063280	BolC09g018000	BraA05g013640	BnA06g0253130
BraA09g015550	AT1G62800	BraA04g022190	BolC04g057200	BraA09g012690	BnA09g0346200
BraA10g020790	AT5G19550	BraA04g022190	BolC04g018900	BraA09g015550	BnA09g0346200
		BraA05g013640	BolC04g018900	BraA02g008260	BnC02g0478340
		BraA05g013640	BolC04g057200	BraA10g020790	BnC02g0478340
		BraA09g012690	BolC04g029290	BraA03g004710	BnC03g0539810
		BraA09g015550	BolC04g029290	BraA04g022190	BnC04g0671000
		BraA09g012690	BolC09g014750	BraA05g013640	BnC04g0671000
		BraA09g015550	BolC09g018000	BraA01g005980	BnC07g0836170
		BraA09g012690	BolC09g018000	BraA03g057120	BnC07g0836170
		BraA09g015550	BolC09g014750	BraA02g008260	BnC08g0861200
		BraA10g020790	BolC02g009850	BraA10g020790	BnC08g0861200
		BraA10g020790	BolC09g052850	BraA09g015550	BnC09g0900940
				BraA09g012690	BnC09g0900940

Table S4 Protein motif sequences of BraASP members

Name	Protein Sequence
Motif 1	LHACAHNPTGVDPTPEQWEQIRQLIRSKNLLPFFDSAYQGFASGSLDTDA
Motif 2	GDWSHIIKQIGMFSFTGLNKEQVDFMTKEYHIYMTSDGRISMAGLSSKTV
Motif 3	QSYAKNMGLYGERIGALSIVCKSEDVARKVKSQLKLVVRPMYSNPPIHGA
Motif 4	GTGSLRVGAEFLAKHYPQRVIFIPNPTWGNHPNIFNLAGLSVEYYRYYDP
Motif 5	PAPEDPILGVTEAYRADPSPVKLNLGVGAYRTEEGKPLVLEVVRKAEQQL
Motif 6	IVATILKBSDMYNDWTIELKGMADRIISMRQQLYEALQAKG
Motif 7	DKEYLPIDGLADFNKLSAKLILGADSPAIKENRVATIQCLS
Motif 8	TRGLDFZGLLEDJGAAPSGAI

Table S5 qPCR primers of *BraASP* genes

Gene Name	Forward 5'-3'	Reverse 5'-3'
BraA01g005980.3C	AGTGGGTGAAAATTGCTGACG	CACTGGCAAAGCCCTGGTAT
BraA02g003960.3C	TTGCTCCGTCATCTCACCG	TGTCAGCGAGGCGAAAATG
BraA02g008260.3C	AAAAAGATAAATTCTCTTCATCC	CGAACGACGTTTGAGAAGACG
BraA03g004710.3C	GCGGATCGCGTGAGCT	AGCTTGAACGAGATGAGCGAA
BraA03g057120.3C	TTAGTCGGGTGACCATGTCG	CGAGAATAGGGTCTGGTGGTG
BraA03g063280.3C	ATTTCTATTTTTCTTCCCAATT	GCAGGAAGGACGTTTGACAAG
BraA04g022190.3C	TCGCCACCTCCTCCTTCTTT	TCTCATCGCCATAATAACTC
BraA05g013640.3C	TCTCCATTAACGCTCCCCCT	CGCCATAGCGCTTTCTTTTT
BraA09g012690.3C	CTCTTGGTTGTCGAGTCTATT	TTATTGAATCAATCGAAAGTGA
BraA09g015550.3C	CACATGACACTCCTTTAAGTAAC	GTTTGACTCAGCGGAAGTGTTG
BraA10g020790.3C	TCACAGCTAATTTATTTGTTTGA	TCCATTAGAGCAAGGATCGCA

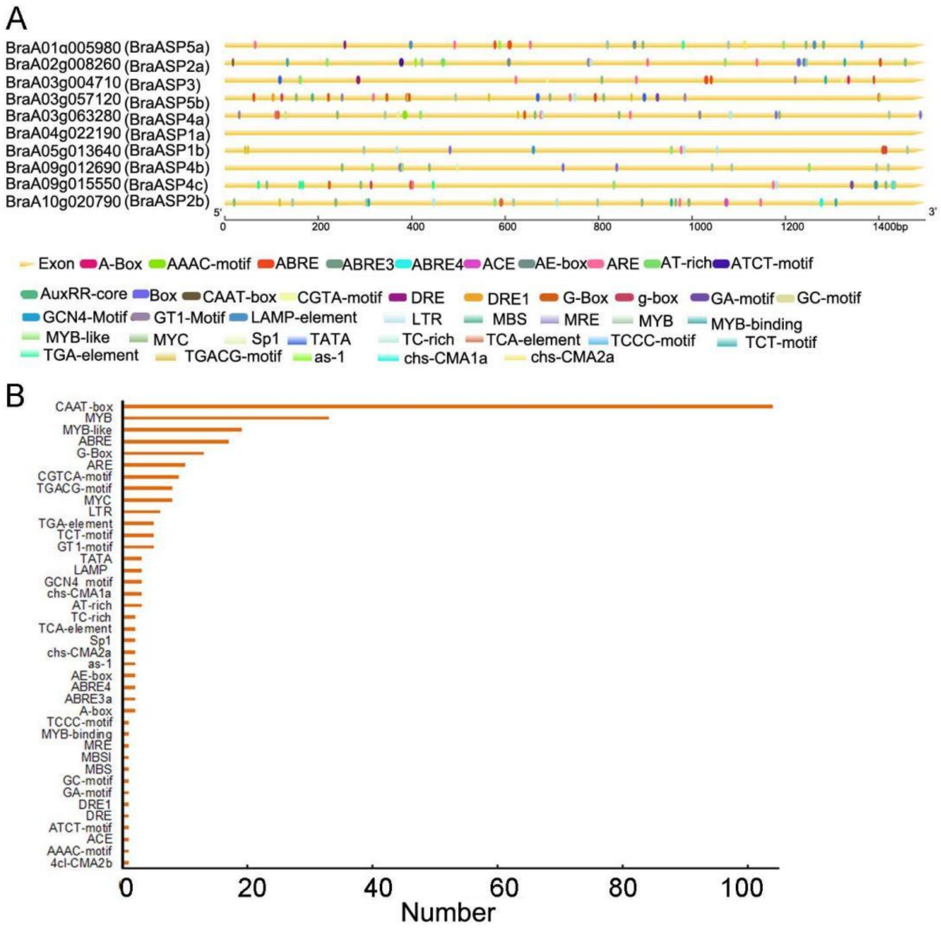

Figure S1 The cis-acting regulatory elements of promoters in *BraASPs*. (A) The distribution of the cis-elements on *BraASP* promoters; (B) The total number of each cis-element on *BraASP* promoters.

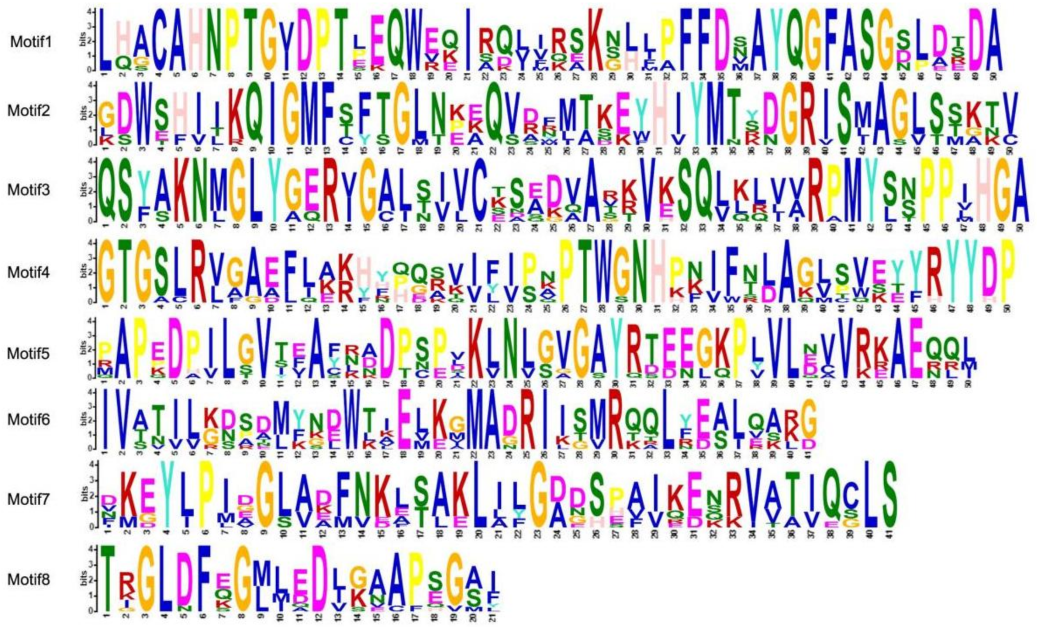

Figure S2 The WebLOGO of protein motif in BraASPs

3'BstEII highlighted in Yellow

Flanking Region(s) highlighted in Green

BstEII are absent in the optimized sequence BrASP

The Optimized (for Brassica rapa) sequence of BrASP

A 333 T 405 C 265 G 282 | GC%: 42.57% | Length: 1285

ATGGCTATGATGGCTAGAACTATCAGAAACTCTGCTTCTAGAAGAACTATGATGATGTCTAGACCTATCTTCGGACTTAGATCTATGTCTTC
TTGGTGGAAGAACGTTGAGCCTGCTCCTAAGGATCCTATCTTGGGTGTTACTGAGGCTTTCCTTGCTGATCCTTCTCCTGATAAGGTTAACG
TTGGAGTTGGAGCTTACAGAGATGATAACGGAAAGCCTGTTGTTCTTGATTGTGTTAGAGAGGCTGAGAGAAGAATCGCTGGAACTTCTTTC
ATGGAGTACCTTCCTATGGGAGGATCTGTTAAGATGGTTGAGGAGACTCTTAAGCTTGCTTACGGAGATAACTCTGAGTTCATCAAGGATAA
GAGAGTTGCTGCTGTTCAATCTCTTTCTGGAACTGGAGCTTGTAGACTTTTCGCTGATTTCCAAACTAGATTCAACCCTGGATCTCAAATCT
ACATCTTCCTTTTCCAACCTGGACCTAACGCTCAAGTTCCTCAAAAGACTTACCATTACTACCATCCTGAGACTAAGGGACTTGATTTCAAG
GGACTTATGGATGATGTTAAGAACGCTCCTGAGGGATCTTTCTTCCTTCTTCATGCTTGTGCTCATAACCCTACTGGAGTTGATCCTACTGA
GGAGCAATGGAGAGAGATCTCTCAACTTTTCAAGGCTAAGAACCATTTCGCTTTCTTCGATATGGCTTACCAAGGATTCGCTTCTGGAGATC
CTGCTAGAGATGCTAAGTCTATCAGAATCTTCCTTGAGGATGGACATCATATCGGAATCTCTCAATCTTACGCTAAGAACATGGGACTTTAC
GGACAAAGAGTTGGATGTCTTTCTGTTCTTTGTGAGAACGAGAAGCAAGCTGTTACTGTTAAGTCTCAACTTCAACAACTTGCTAGAGCTAT
GTACTCTAACCCTCCTCTTCATGGAGCTCAAATCGTTTCTACAATCCTTGGTGATCCTGAGCTTAAGTCTCTTTGGCTTAAAGAGGTTAAGA
TCATGGCTGATAGAATCATCGGAATGAGAACTACTCTTAGAGAGTCTCTTGAGAAGCTTGGATCTCCTCTTTCTTGGGAGCATGTTACTAAG
CAAATCGGAATGTTCTGTTACTCTGGACTTACTCCTGAGCAAGTTGATAGACTTACTTCTGAGTACCATATCTACATGACTAGAAACGGAAG
AATCTCTATGGCTGGAGTTACTACTGGAAACGTTGGATACCTTGCTAACGCTATCCATGAGGTTACTAAGTCTTCTTGAATTGGTGACC

Figure S3 The codon optimization sequences of *BraAsp1b*
